# Gold-Induced Structural Switch of Cys34 in Albumin: Comparison of Auranofin With Aurothiomalate

**DOI:** 10.1155/MBD.1994.527

**Published:** 1994

**Authors:** John Christodoulou, Peter J. Sadler, Slan Tucker

**Affiliations:** 1 Department of Chemistry Birkbeck College University of London 29 Gordon Square London WC1H 0PP United Kingdom


					GOLD-INDUCED STRUCTURAL SWITCH OF CYS34 IN ALBUMIN:
COMPARISON OF AURANOFIN WITH AUROTHIOMALATE
John Christodoulou, Peter J. Sadler and Slan Tucker
Department of Chemistry, Birkbeck College, University of London,
29 Gordon Square, London WC1H 0PP, UK
Albumin, a single-chain protein of ca. 580 amino acids (66.5 kDa), is the major protein in blood serum
with a concentration of ca. 0.63 mM. The protein .consists of three largely a-helical structurally-similar
domains, each of which contains two sub-domains [1]. It plays a central role in the molecular
pharmacology of gold drugs used for the treatment of rheumatoid arthritis, carrying ca. 80% of
circulating gold in the body.
The primary binding site for Au(I) on albumin is known to be at the sulfur of Cys34 in domain [2]. We
have investigated the structural changes induced, in bovine and human albumin by the oral drug
auranofin, its deacetylated metabolite, Et3PAuCI, the injectable drug aurothiomalate, and related
thiols, using NMR spectroscopy.
In reactions of albumin with auranofin and tetraacetylthioglucose, cystine was detected as a product,
whereas for deacetylated auranofin, thioglucose and glutathione, mixed disulfides with cysteine were
produced. These reactions appear to involve reductive deblocking of albumin disulfides via thiol-
disulfide interchange reactions [3].
The behavior of the His1 1H NMR resonance of His3 suggests that albumin exists in two distinct
structural forms in solution dependent on whether the side-chain of Cys34 is buried or exposed.
Modification of Cys34 by gold binding, disulfide formation or other oxidation, switches the conformation
to the exposed form [4]. Such a structural change may influence the metabolism of albumin in vivo, as
well as its other binding properties.
The buried-exposed conformational switch of Cys34 induced by aurothiomalate shows an interesting
ionic strength dependence. In this case the gold -induced switch may require a concomitant anion-
induced structural change in the protein. This interdependence may influence the uptake and transport
of aurothiomalate by albumin in vivo.
We thank the Wellcome Trust, EPSRC, MRC and Delta Biotechnology for their support for this work.
References
[1] D.C. Carter and J.X. Ho, Adv. Prot. Chem. 1994, 45, 153-203
[2] C.F. Shaw III, Comm. Inorg. Chem. 1989, 8, 233-267.
[3] O.M. Ni Dhubhghaill, P.J. Sadler and A. Tucker, J. Am. Chem. Soc. 1992, 114, 1117-1118.
[4] J. Christodoulou, P.J. Sadler and A. Tucker, Eur. J. Biochem. 1994, in press.
527

				

